# A socio-structural approach to preventing injection drug use initiation: rationale for the PRIMER study

**DOI:** 10.1186/s12954-016-0114-1

**Published:** 2016-09-15

**Authors:** Daniel Werb, Richard Garfein, Thomas Kerr, Peter Davidson, Perrine Roux, Marie Jauffret-Roustide, Marc Auriacombe, Will Small, Steffanie A. Strathdee

**Affiliations:** 1 Division of Global Public Health, Department of Medicine, University of California San Diego, San Diego, USA; 2 Urban Health Research Initiative, British Columbia Centre for Excellence in HIV/AIDS, Vancouver, Canada; 3 INSERM, UMR_S 912, Sciences Economiques & Sociales de la Santé et Traitement de l’Information Médicale (SESSTIM), F-13385 Marseille, France; 4 Inserm U988/CNRS UMR 8211, Ecole des Hautes Etudes en Sciences Sociales, Université de Paris Descartes, Paris, France; 5 SANSPY/CNRS USR 3413, Université de Bordeaux, Bordeaux, France; 6 Division of Global Public Health, University of California School of Medicine, University of California San Diego, 9500 Gilman Drive, La Jolla, CA 92093-0507 USA

**Keywords:** HIV prevention, Injection initiation, Natural history of injecting, Street youth, People who inject drugs, Multi-site study

## Abstract

**Background:**

Injection drug use remains a primary driver of HIV and HCV-related harms globally. However, there is a gap in efforts to prevent individuals from transitioning into injecting. People who inject drugs (PWID) play a key role in the transition of others into injecting, and while behavioral interventions have been developed to address this phenomenon, socio-structural approaches remain unexplored. To that end, we hypothesize that certain interventions designed to reduce injecting-related risk behaviors may also reduce the risk that PWID expose and introduce others into injecting. Identifying the preventive potential of existing interventions will inform broader efforts to prevent injecting and related harms.

**Methods:**

The Preventing Injecting by Modifying Existing Responses (PRIMER) study is a multi-country mixed methods study with an aim to investigate whether specific interventions (e.g., opioid substitution therapy, supervised injection facilities, stable housing, incarceration environments) and related factors (e.g., public injecting and gender) influence the likelihood that PWID initiate others into injecting. This study will (1) investigate the PWID participation in injection initiation; (2) identify factors influencing the risk that PWID expose others to or facilitate injection initiation; (3) describe drug scene roles that increase the risk of PWID facilitating injection initiation; and (4) evaluate the impact of structural, social, or biomedical interventions on the risk that PWID facilitate injection initiation. It does so by pooling observational data from cohort studies of PWID in six cities: Vancouver, Canada; San Diego, USA; Tijuana, Mexico; Paris, Marseille, and Bordeaux, France.

**Results:**

Team members are conducting a prospective, multi-site study of PWID (*n* = 3050) in North America and France that includes quantitative and qualitative data collection through four separate cohort studies of PWID (San Diego, STAHR II; Tijuana, El Cuete IV; Vancouver, V-DUS; Bordeaux, Marseille, Paris and Strasbourg, COSINUS).

**Conclusions:**

PRIMER is the largest study of injection initiation to date and the first to investigate structural approaches to preventing injection drug use initiation. Findings have the potential to inform the development and scale up of new and existing interventions to prevent transitions into injecting.

**Trial registration:**

Preventing Injecting by Modifying Existing Responses (PRIMER), NIDA DP2-DA040256-01.

## Background

Injection drug use remains a primary source of HIV- and hepatitis C (HCV)-related harms [1], with a recent estimate suggesting that 15.9 million people inject drugs globally. Among this population, three million (19 %) are believed to be HIV-positive [1], while ten million people who inject drugs (PWID) are estimated to be HCV-positive [2], with prevalence among PWID populations believed to exceed 60 % in 37 countries [2]. The median age of injection initiation globally is 19, indicating that more than half of PWID initiate injecting as adolescents or young adults [3]. Of concern, data also suggest that the window of opportunity to prevent blood-borne virus transmission among new injectors is limited, given that the majority of incident HCV and hepatitis B virus cases occur within 1 year of initiation [4], which may be due to elevated incidence of risk behaviors such as syringe sharing among recently initiated injectors [1]. The increased addictive potential of injecting also creates challenges in preventing risky drug-related behaviors [5]. As such, injecting remains a primary driver of HIV and viral hepatitis epidemics worldwide. Recent increases in prescription opioid misuse in North America may be contributing to a heightened risk of injection initiation [6], given that the use of prescription opioids such as oxycodone has been shown to be associated with a heightened risk of initiating injecting [7].

Studies to date suggest that the majority of injection initiation events are facilitated, either directly or indirectly, by PWID [8–19]. For example, among a sample of street youth who reported injecting drugs in Vancouver, Canada (*n* = 369), almost three quarters reported being initiated by other injectors [10]. Among a cohort of PWID in Tijuana, Mexico (*n* = 1052), only 11 % reported initiating injection alone [20]. In France, among the PWID enroled in a cross-sectional study in five cities (*n* = 1077; Lille, Strasbourg, Paris, Bordeaux, and Marseille), 83 % of participants reported having been initiated by another person [21]. A qualitative study undertaken among a sample of newly initiated PWID in New York City (*n* = 54) also found that decisions to begin injecting were influenced by previous exposure to injecting and that initiates actively sought out PWID to assist with initiation [12]. Determining the local factors driving such trends is critical in developing adaptable and effective interventions to prevent epidemics of injecting.

In particular, preventing injection initiation requires an understanding of individual roles within drug scenes that may place PWID at higher risk of initiating others. For example, “hit doctors” (i.e., individuals paid with money or drugs to inject others) may be asked for injection assistance by customers at the time they purchase their drugs. Hit doctors are often present in and around risky injecting environments such as shooting galleries and public injecting venues [22] and may be sources of injecting education [23], placing them at higher risk of being approached for injection initiation assistance. Importantly, the context of initiation may also differ based on the gender of both initiates and initiators, with female initiates being more likely to be injected by older male PWID, who are often intimate partners [24]. These findings suggest that PWID facilitate transitions to injecting in multiple ways: by exposing injection-naïve drug users to injecting [10], by providing injecting education [12], and by directly injecting initiates [15]. While a minority of PWID report facilitating injection initiation events, those who do may initiate numerous individuals. Among a sample of Californian PWID (*n* = 605), for example, 35 % (*n* = 214) reported never initiating others into injecting, with a total of 3271 individuals initiated into injecting [15]. Similarly, while only 17 % (*n* = 55) of a sample of Australian PWID (*n* = 324) reported initiating others, they initiated 128 new injectors [16].

Reducing the exposure of injection-naïve drug users to injecting practices is therefore likely to reduce their risk of transitioning into injecting. Interestingly, data suggest that neighborhoods with high levels of public injecting are sites of increased population mixing between PWID and non-injectors [14]. For example, non-injecting street youth in Vancouver residing in the downtown eastside, which is characterized by high rates of public injecting, reported a cumulative incidence of initiation after 3 years that was over twice that reported by non-injecting street youth residing elsewhere in the city [14]. Evidence also suggests that the deployment of intense policing of street-level drug markets is likely to intensify risky injection practices [25] and may also increase the exposure of non-injectors to PWID by dispersing drug markets spatially [26] or by increasing the risk that PWID will inject in public or semipublic venues, a phenomenon that has been widely observed across a range of settings [27–35]. The incarceration of PWID likely also increases the risk that they may expose or initiate non-injectors to injecting, given the potential for population mixing in prison [17] (this is of particular interest given recent moves towards de-incarceration of PWID in California and Mexico [36]).

Although there is strong evidence that PWID play a key role in the initiation of other individuals into injection drug use [13], there are few interventions that prevent injection initiation by focusing on the role of PWID in this practice [13]. To date, these have by and large employed behavioral approaches that seek to directly alter the individual-level decision-making of PWID at risk of initiating others into injecting. There is therefore a dearth of research and intervention development focused on modifying the social and structural factors that in turn influence PWID to initiate others.

We hypothesize that the interventions that reduce the frequency of public injecting may have the secondary benefit of preventing injection initiation by limiting the exposure of non-injectors to injecting. For instance, opioid substitution therapies (OST) including methadone and buprenorphine maintenance therapy, supervised injection facilities (SIF), and stable housing for PWID have all been shown to reduce the risk of PWID injecting in public venues [37]. If public injecting increases the exposure of non-injectors to injecting, the provision of such interventions may therefore reduce the risk that PWID expose injection-naïve drug users to injecting, thereby decreasing the latter’s risk of injection initiation. Within settings characterized by population mixing between PWID and non-injectors, there is often a “code” or ethic among established injectors discouraging the initiation of others, but facilitating initiation may be difficult to avoid for PWID who may be offered money or drugs to facilitate initiation events [9]; this may be particularly acute in the context of individuals with substance use disorders who lack resources to purchase drugs or have inadequate access to addiction treatments such as OST.

Given the gaps in knowledge regarding injection initiation, as well as the lack of effective interventions focused on this practice, a 5-year multi-country study has been developed to investigate structural, social, and biomedical approaches to preventing injection initiation and to identify individual pathways and drug scene roles associated with facilitating initiation. Herein, we describe the methodology as well as the participant and site characteristics.

## Methods

### Preventing Injecting by Modifying Existing Responses: PRIMER

The Preventing Injecting by Modifying Existing Responses (PRIMER) is a US National Institute on Drug Abuse-funded study (DP2- DA040256-01; PI: Werb) seeking to determine whether interventions effective in reducing public injecting can reduce the initiation of others into injecting and to then employ these findings to develop a socio-structural interventional approach to preventing injection drug use.

### Conceptual framework

**Fig. 1:**
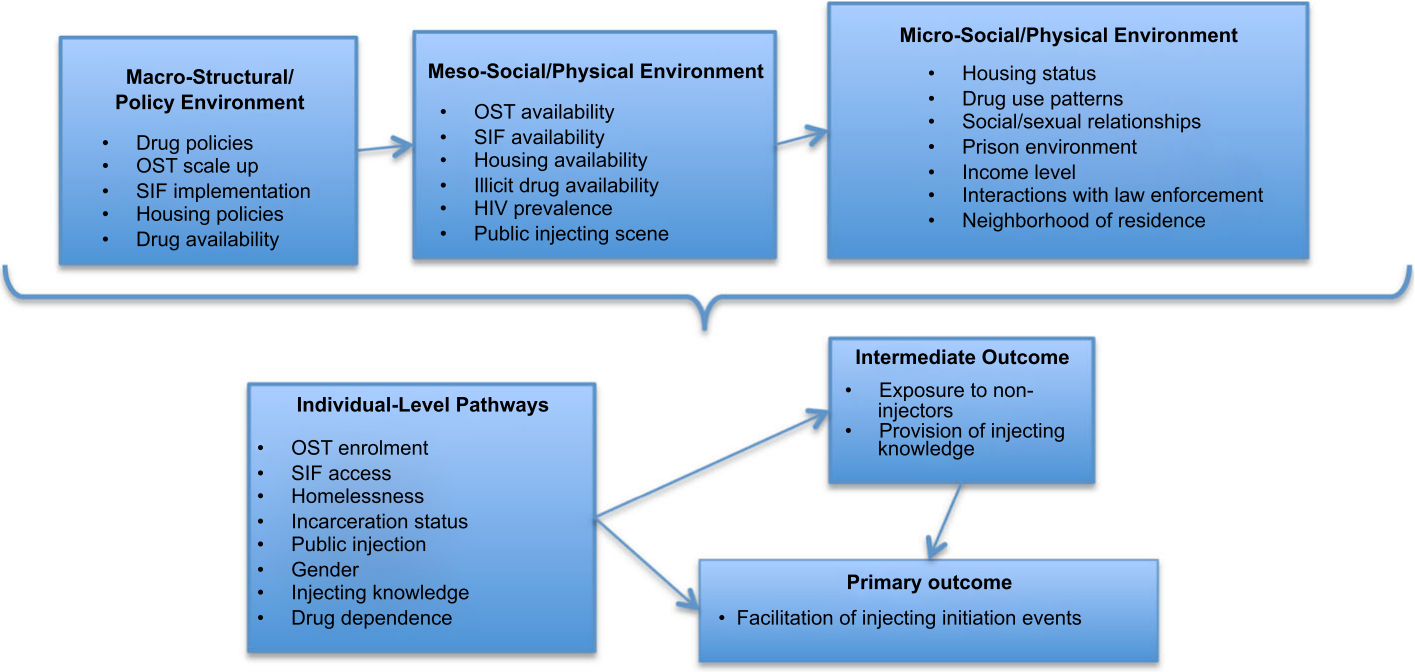
Conceptual framework to investigate injection initiation events facilitated by established injectors

Rhodes’ risk environment framework allows for the investigation of the social and structural environment experienced by PWID and an identification of how this may limit the capacity of PWID to avoid drug-related harm [38, 39], including high risky injection practices such as exposing injection-naïve drug users to injecting behaviors [40]. The risk environment framework also allows for an examination of how interactions between social, structural, physical, and political/economic influences operating at micro, meso, and macro levels of an individual’s environment may shape their local drug scene and behavior. This is particularly useful in considering the intersection of individual-level behaviors—in this case, exposing or initiating others into injecting—with a range of social, structural, and economic factors operating at multiple levels. PRIMER will examine the interaction of individual-level factors such as OST enrolment, housing status, spatial proximity to non-injectors, income level, and level of drug dependence; meso-level factors such as the presence of shooting galleries, public injecting settings, SIF implementation, OST programs, and drug use patterns in local PWID populations; and macro-level factors such as the type of drug policies and housing policies in place, along with the availability of illicit drugs (Fig. 1). This approach is consistent with interventional approaches that consider points of maximum effect and the interaction of factors, at the structural level (macro), environmental level (meso), and individual level (micro) [41].

### Approach

The PRIMER study has four major aims across two phases. Phase I consists of mixed methods data collection and analysis. Phase II consists of the evaluation of an interventional approach to prevent injection initiation informed by the findings of phase I. Aim 1 is to determine the prevalence and characteristics of PWID participation in injecting initiation across multiple study sites. Aim 2 is to assess factors potentially influencing the risk that PWID facilitate injecting initiation. Primary factors of interest are as follows: enrolment in OST, SIF access, stable housing, a history of incarceration, public injection, and gender. Aim 3 is to qualitatively investigate individual pathways and roles that increase the risk of PWID facilitating injecting initiation. This involves exploring local social norms across study sites related to injecting others among PWID through in-depth interviewing. We will specifically investigate gender within the context of injecting initiation events, with a focus on intimate partnerships. We will also explore the role of injecting environments (e.g., shooting galleries, jails/prisons, SIF, public vs. private settings) and drug scene roles (e.g., hit doctors, drug dealers, sex workers) that may heighten the risk that PWID facilitate injecting initiation. Finally, participants’ own initiation events will be explored to determine how they influence subsequent decisions to initiate others. Finally, aim 4 (carried out in phase II) is to test the impact of an existing structural or biomedical intervention on the risk that PWID will facilitate injecting initiation. Based on the findings generated through the exploration of aims 1–3, we will evaluate the implementation and/or scale-up of an interventional approach addressing the factors that most greatly influence the risk that PWID initiate others into injecting.

In phase I, quantitative and qualitative data on involvement in injection initiation collected from PWID aged 18 years and older in three longitudinal cohort studies in North America and one longitudinal cohort study in France are being pooled and analyzed. These data are being used to determine the prevalence and characteristics of PWID participation in injection initiation across multiple study sites, to assess a range of factors at multiple levels that potentially influence the risk that PWID facilitate injection initiation, and to investigate individual pathways and roles that increase the risk of PWID facilitating injection initiation. This consists of both quantitative and qualitative data collection to explore local contexts and social norms related to PWID’s initiation of others into injecting in each study site.

### Study sites

While we hypothesize that structural, social, and biomedical interventions to reduce injection-driven HIV and HCV transmission risk might also reduce the risk that PWID facilitate injection initiation, we anticipate that the impact of these interventions will be mediated by unique local characteristics in different settings and among discrete populations of PWID. PRIMER therefore includes longitudinal data from four cohort studies of PWID aged 18 and older located in urban settings in the USA (PI Dr. Richard Garfein, *Study to Assess Hepatitis C Risk* (STAHR II); San Diego, CA; NIH R01-DA031074); Tijuana, Mexico (PI Dr. Steffanie Strathdee, *Proyecto El Cuete IV* (ECIV); Tijuana; NIH R37-DA019829); Vancouver, Canada (PI Dr. Thomas Kerr, V-DUS; Vancouver; NIH R01-DA011591); and France (PIs Pr. Marc Auriacombe and Drs. Marie Jauffret-Roustide and Perrine Roux, *COhorte pour l’évaluation des facteurs Structurels et INdividuels de l’USage de drogues* (COSINUS); Bordeaux, Marseille, Paris, and Strasbourg; INSERM/MILDECA C14-26).

### Quantitative data collection

Identical questions on the involvement of PWID in injection initiation were seeded into cohort survey instruments in September 2014 and have been integrated into broader data collection protocols. Questions solicit data on participants’ experiences with initiating others into injecting, the reasons for doing so, the relationship between initiates and initiators (including their gender), and the self-perceived likelihood that participants would initiate others in the future. These questions form the basis of the project’s quantitative data collection and will allow for investigations of the lifetime history and recent experiences (i.e., past 6 months) of PWID’s facilitation of injection initiation. Importantly, these cohort studies all seek to investigate HIV risk behaviors among PWID residing in urban centers, and their survey instruments share important similarities that facilitate cross-site comparisons. Additionally, the STAHR II and El Cuete IV study instruments were intentionally designed for cross-study comparisons [42]. Prior to data pooling, each participant’s data is assigned a unique identifier (including a site locator) to avoid duplication or misclassification.

Recruitment for all participant cohort studies is via targeted sampling methods that include outreach activities, snowball sampling methods, and self-referral. Potential participants are invited to participate in the study, and in the case of STAHR II, El Cuete IV, and VIDUS II, those individuals who consent are asked to provide a specimen for rapid HIV testing (COSINUS participants do not undergo serologic testing). Outreach staff, including interviewers, frontline personnel, and field staff, as well as nurses, are, in all cases, familiar with local neighborhoods and the social structural contexts of injection drug use and have extensive experience in recruiting and following up with participants from local PWID networks. The field offices employed for participant interviews and interactions are designed as low-threshold, easily accessible, and friendly environments, with staff trained to be courteous and respectful of participants. In all studies, staff involved—and particularly research coordinators and outreach staff—have extensive experience working with addicted and marginalized individuals. Study interviewers are trained in survey administration, techniques to ensure trust and rapport with participants, and methods to ensure confidentiality. All interviews take approximately 60–90 min and are conducted in secure field offices and locales PWID are known to frequent. All follow-up interviews occur at 6-month intervals.

### Statistical analytic plan

Our primary analytic approach employs generalized linear mixed models (GLMM), which addresses problems of attrition, missing data, variable timing of subject visits, and other unintended violations of design. Temporal correlations between subject responses are assumed using an autoregressive structure, with subject-specific variances and correlations assumed to decline exponentially over time. Importantly, this approach also allows for the inclusion of study site as a nesting factor. To evaluate the impact of socio-structural factors on the risk that PWID facilitate injection initiation, GLMM models will be fitted with reporting recent (i.e., past 6 months) injection initiation facilitation as the outcome. This is defined using the following survey item: “In the last 6 months, have you helped anybody inject who had never injected before?” (yes vs. no). The primary independent variables of interest are defined as follows: recent (i.e., past 6 months) OST enrolment, recent attendance at a SIF (Vancouver only), recent housing stability, recent incarceration, recent public injecting, and gender. To investigate how local settings impact risk, the effect of the independent variables of interest on facilitating injection initiation is being evaluated separately for each location. We anticipate a range of confounders which may influence the association between our outcome variable and independent variables of interest. While these are likely to vary across sites, we anticipate testing the impact of the following: intimate partnerships, migration, stigma, HIV status, mental health, health-care utilization, level of drug dependence, income level, ethnicity, and attitudes regarding social norms around injecting.

### Qualitative data collection

In-depth qualitative interviews will be conducted among participants from all cohort studies. Sampling is purposive and based on either reporting facilitated injection initiation in quantitative interviews or with reporting no injection initiation but having a quantitative “profile” (i.e., identical answers to a set of relevant cohort survey items) consistent with other participants that report facilitating injection initiation. This approach allows for qualitative comparisons between individuals who do and do not initiate others into injecting despite a profile that suggests that they are at risk; this will facilitate the development of targeted interventional approaches. We will weight our sampling based on the experience with the independent variables of interest. For example, to ensure recruitment of hit doctors and drug dealers, we will recruit participants who indicate in quantitative surveys that they have initiated others into injecting and have also undertaken these drug scene roles. We will seek to recruit 25–35 participants from each cohort. In qualitative interviews, we will specifically investigate gendered dynamics within the context of injection initiation events, with a focus on intimate partnerships. We will also explore the role of injecting environments (e.g., shooting galleries, jails/prisons, SIF, public vs. private settings) and drug scene roles (e.g., hit doctors, drug dealers, sex workers) that may heighten the risk that PWID facilitate injection initiation. Finally, we will explore the participants’ accounts of their own initiation events to determine how these influence subsequent decisions to initiate others.

### Qualitative analysis

Grounded theory will be used as the overarching conceptual approach to qualitative data collection and analysis [43, 44]. Grounded theory is founded on an approach that assumes that one’s communication and actions express a meaning that is both individually held and shared socially [43]. As such, grounded theory allows for the identification and delineation of core processes or assumptions that underlie specific individual actions or social interactions, with the ultimate aim of identifying a coherent model for the individual and social actions observed. In the context of identifying individual pathways toward injection initiation, as well as the social relationships that may propel an individual to begin injecting, grounded theory will be employed to collect and analyze data in order to develop a model of injection initiation grounded in social interaction.

### Mixed methods analytic plan

A parallel mixed methods design is being employed, which is followed by a meta-inference process wherein results of the quantitative and qualitative analyses are being synthesized [45]. This approach represents an “ethno-epidemiological” research design, as it integrates targeted qualitative research in relation to epidemiological cohort studies, and greater understanding of the lived experience of PWID can add a deeper social dimension to traditional quantitative measures regarding specific outcomes (in this case, injection initiation), as has been done previously [46–48]. Because effect sizes for each independent variable of interest will differ across study sites, qualitative findings are being used to determine which causal pathways, individual roles, or relationships shape these differences. The meta-inference process is iterative and, as data are collected and triangulated, will refine our understanding of the factors driving PWID to facilitate initiation.

### Phase II intervention development strategy

The type of intervention, or combination, to be tested in phase II will be determined after the analysis of findings from phase I. This tailored approach extends to the selection of the most appropriate setting for evaluation. As outlined above, important differences exist across study sites, with interventions being scaled up or implemented during the study period; the situation “on the ground” in each study site, in combination with phase I findings, will therefore determine which site is ultimately selected for evaluation and whether an existing, modified, or novel intervention is evaluated. While dependent on findings, we have identified methodological approaches appropriate for intervention testing. Community randomization, with experimental and delayed onset control arms, may be appropriate to test interventions such as subsidized OST or SIF. Interrupted time-series analyses may also be suitable to test widespread policy change (e.g., de-incarceration).

Discrete HIV epidemics are driven by local dynamics, and we therefore anticipate that the findings generated in phase I will likely demonstrate that a combination of socio-structural factors heighten the risk that PWID facilitate injection initiation, and thereby contribute to a higher population-level risk of HIV transmission. As above, relevant potential factors vary greatly by study setting and have implications for the impact and optimal combination of interventions. For example, in Vancouver, a combination of OST and SIF access may reduce the risk that PWID initiate others; in Tijuana, access to stable housing and de-incarceration may be found to most greatly reduce risk. In San Diego, with low OST enrolment and high levels of homelessness, and a rapid move towards de-incarceration [49], multiple potential interventional points exist. In France, OST coverage is very high (70–80 %) (and has been accompanied by a reduction in the overall incidence of sharing behaviors and HIV and HCV transmission in local contexts [50, 51]); this provides an opportunity to determine how scale up of OST may impact injection initiation risk.

### Study sites

Table 1 provides relevant study site and baseline participant characteristics for each cohort study included in PRIMER. These data do not include COSINUS sample data given that data collection for this cohort has not yet started. We also present data from the VIDUS II cohort, which is being transitioned into the V-DUS study in 2016 in order to integrate cohorts of PWID and street-involved youth, some of whom are injection-naïve. The estimated sample sizes for each cohort are as follows: STAHR II, *n* = 575; Proyecto El Cuete, *n* = 750; V-DUS, *n* = 1735; and COSINUS, *n* = 680. As such, the pooled sample size for PRIMER will approach 3740 participants, though we caution that missing data, loss to follow-up, and incompatibility between variables in cohort questionnaires are likely to reduce this overall number. As can be seen in Table 1, female participants comprise between 26 and 38 % of the cohort participants, and HIV prevalence at baseline ranged from 0–10 % (note that only HIV-seronegative PWID participants are eligible for inclusion in VIDUS II, though V-DUS will not include this restriction). Overall, the prevalence of lifetime history of accessing OST was highest in Vancouver (53 %) and lowest in Tijuana (28 %). In Vancouver, 23 % of participants also reported accessing OST in the past 6 months. We anticipate that reported OST access will be higher among COSINUS participants given much higher levels of OST scale up in France [52].

**Table 1:** Baseline cohort characteristics

Study	Location	Number	Eligible age range	Study period	OST uptake	SIF present	Homeless in the past 6 months (%)	Incarceration	HIV+ in sample (%)	Female (%)
Proyecto El Cuete IV	Tijuana, Mexico	750	18+	2010–2020	28 % ever	No	20	71 % ever	5	38
STAHR II	San Diego, USA	575	18+	2012–2016	23 % ever	No	55	78 % ever	10	26
V-DUS^a^	Vancouver, Canada	1735	18+	1996–2020	53 % ever	Yes	5	19 % ever	0^d^	31
COSINUS^b^	Paris, Marseille, Bordeaux, and Strasbourg, France	680	18+	2016–2018	–	Yes^c^	–	–	–	–

^a^Refers to current VIDUS II sample (set to merge into V-DUS in 2016)

^b^Data collection to begin in Fall 2016

^c^SIF likely to be implemented in Paris and Strasbourg in Fall 2016

^d^All VIDUS II participants are HIV-negative at baseline

### Ethics, consent, and permissions

The study was provided ethical approval by the Institutional Review Board of the University of California, San Diego School of Medicine (UCSD IRB 150866). We have also obtained consent to publish and report individual participant data.

## Results and discussion

Injection drug use remains one of the key drivers of HIV and HCV epidemics worldwide. And yet, very few interventions have proven effective in preventing individuals from initiating injection drug use. Although experts support efforts to reduce injecting exposure through individual-level behavioral change among PWID [13], structural approaches to reducing the risk that PWID expose and initiate others into injecting have not yet been adequately developed. While preliminary data strongly suggest that PWID play a central role in facilitating injection initiation, a critical gap in programing to address this practice remains.

PRIMER, which seeks to harness the preventive potential of existing structural, social, and biomedical HIV interventions, is, to our knowledge, unique in addressing this gap in public health programing. This is highly relevant given the potential for increases in injection drug use presented by the rising prevalence of prescription opioid misuse in North America [6], the emergence and expansion of epidemics of injecting in West and Sub-Saharan Africa and Asia [53], and the potential impact of widespread OST coverage on mitigating injection initiation risk. We also anticipate that the mixed methods analytic approach we employ will further confirm gender and drug market roles (e.g., hit doctors) as uniquely contributing to the risk of injection initiation posed by PWID [11, 12]; this may allow for the development of gender-specific interventions to prevent injection initiation. A peer-based intervention tailored to other specific subpopulations may be required to reduce the risk that certain PWID initiate others; these might include Break the Cycle or the “heroin sniffers” projects (both behavioral interventions that seek to build resilience among PWID to avoid initiating others) [13], which are currently being studied in combination in a research trial being carried out in New York City, USA, and Talinn, Estonia [54]. Relatedly, the potential that hit doctors may be more likely to initiate others might also require adaptation of existing injection education interventions [55]. These models generally involve peer educators who disseminate information about safer injection practices [56]; as such, they could be used to also disseminate strategies to discourage injection initiation, as well as to provide information to PWID on accessing existing interventions such as OST and SIF.

With respect to the selection of study sites, the decision was based primarily on a recognition, as above, that patterns of injection drug use initiation are driven by unique local dynamics. We therefore sought to include sites that represented diverse economic, legal, policy, and cultural settings. We note that while these site characteristics can be identified a priori, other important factors that may influence patterns of injecting initiation—particularly stigma, drug use trends, discrete social networks, and social norms around injecting—will be identified during the study and will be used to inform intervention development. Specifically, Vancouver has a large, densely located PWID population, with a high but declining prevalence of HIV [57]. In the mid-1990s, drug use patterns among PWID shifted from heroin to cocaine injection, which was undertaken with higher frequency, resulting in higher rates of contaminated syringe sharing and a subsequent increase in HIV incidence [58]. Subsequently, a range of harm reduction and ancillary services were implemented or scaled-up, such as OST and a limited number of SIFs [57], including a peer-run unsanctioned SIF that allows assisted injection [59]. The existing of a peer-run SIF may allow for intervention among individuals such as hit doctors who may more frequently engage in injection initiation events [60].

San Diego is characterized by a PWID population estimated most recently at approximately 20,000 (standard deviation 12,000) [61] highly dispersed across the county. PWID in San Diego report primarily injecting with heroin and crystal methamphetamine [62]. HIV prevalence among PWID is estimated at 4 % [42], and access to services such as needle exchange and OST is low. Importantly, California has had among the most stringent criminal justice responses to drug use and possession since 1994 and has experienced extreme prison overcrowding [49]. However, the passage in November 2014 of Proposition 47, a statute that reclassifies non-violent drug crimes from felonies to misdemeanors, will result in the release of potentially 10,000 currently incarcerated individuals and is estimated to contribute to 40,000 fewer felony convictions annually [49]. This site therefore presents an opportunity to assess the impact of a large-scale structural intervention on injection initiation risk, in a setting currently characterized by high incarceration rates.

Tijuana has a densely situated local PWID population (estimated *N* = 10,000), located primarily within the Zona Norte neighborhood (adjacent to the US-Mexico border) [63], with a high level of population mixing between PWID and non-injectors as a result of deportation from the USA [64, 65]. HIV prevalence is low (i.e., 4 %) [42], and addiction treatment and harm reduction services are under-resourced [66]. However, the passage of a partial drug decriminalization law in 2009 has seen a gradual shift towards increased addiction treatment scale-up (including OST) in Tijuana [67]. Accordingly, the number of OST clinics has increased slightly, while shifting policing strategies are beginning to impact the rate of diversion of PWID towards addiction treatment services [36]. The STAHR II and El Cuete IV study instruments were deliberately designed to be identical to allow cross-border comparisons of PWID in these adjacent cities [42].

France (Bordeaux, Marseille, Paris, and Strasbourg) is unique in having high levels of OST scale-up, with an estimated 180,000 individuals reporting OST access [50] (in specific sites, estimates of OST access among PWID exceed 70 %) [68, 69]. Further, despite delays, SIF are expected to be implemented in at least one study site (i.e., Paris) during the project period [70, 71], providing an opportunity to investigate how a combination of high OST coverage and SIF access may impact PWID risk of facilitating initiation.

### Strengths and limitations

The employment of existing NIDA-funded cohort study mechanisms ensures that data are of high quality and that sufficient statistical power for analyses is obtained. The focus on multiple factors (i.e., OST enrolment, SIF access, housing status, incarceration, public injecting, and gender), in isolation and in combination, also allows for a high degree of adaptation to local contexts. It also provides a foundation for future modeling of the broader impact of these interventions on injection drug use prevention in a variety of settings.

This project, though, has limitations. First, it is likely that participation in injection initiation may be underreported by PWID given the stigma associated with this behavior [12], which may result in threats to statistical power or misclassification of data. However, the large estimated sample size (*n* = 3760) provides a large amount of statistical power. Second, there may be discrepancies in data collection across sites that may limit our analyses (i.e., ECIV and STAHR II both employ computer-assisted personal interviewing (CAPI), while V-DUS does not (nor will COSINUS), which may result in misclassification given that recall of risk behaviors may differ between cohort participants interviewed via CAPI and those interviewed using other techniques) [72–74]. However, as noted, all study questionnaires are highly comparable, and we are undertaking extensive qualitative data collection as part of a mixed methods approach to obtain a more contextualized and detailed understanding of the roles, social influences, and contexts that shape PWID risk of facilitating injection initiation. Third, while study samples may not be generalizable, data from multiple settings allows for an understanding of how socio-structural factors in combination impact the risk of injection initiation and, ultimately, of HIV transmission. Fourth, challenges with scale-up exist with all interventions. However, team members have extensive experience in implementing and evaluating cutting-edge HIV interventions [75] and have also developed institutional and multi-sectoral partnerships to support such activities in all study sites.

## Conclusions

There is a critical gap in the global development and scale-up of evidence-based responses to preventing injection initiation. Given the contribution of injecting to the risk of transmission of HIV and other blood-borne diseases, it is imperative that resources be allocated towards identifying effective approaches to preventing injection drug use, rather than focusing solely on injection-related health harms. To ensure maximum effectiveness, any preventive approach must also be both adaptable to a range of settings and easily integrated into the suite of current public health programing designed to address the needs of drug-using populations [76]. These are the collective goals of PRIMER, and we anticipate that study findings will help support the development and evaluation of a comprehensive and scalable approach to reducing the incidence of injection-related morbidity and mortality experienced in a wide range of settings internationally [1, 77–79].

## Abbreviations

CAPI: Computer-assisted personal interviewing
COSINUS: COhorte pour l’évaluation des facteurs Structurels et INdividuels de l’USage de drogues
ECIV: Proyecto El Cuete IV
HCV: Hepatitis C virus
HIV: Human immunodeficiency virus
OST: Opioid substitution therapy
PRIMER Study: Preventing Injecting by Modifying Existing Responses Study
PWID: People who inject drugs
SIF: Supervised injection facility
STAHR II: Study to Assess Hepatitis Risk II
V-DUS: Vancouver Drug Users Study
